# Gut Microbiota Dysbiosis and Its Impact on Type 2 Diabetes: From Pathogenesis to Therapeutic Strategies

**DOI:** 10.3390/metabo15060397

**Published:** 2025-06-12

**Authors:** Yonghua Yu, Yilan Ding, Shuangyuan Wang, Lei Jiang

**Affiliations:** 1 Department of Endocrine and Metabolic Diseases, Shanghai Institute of Endocrine and Metabolic Diseases, Ruijin Hospital, Shanghai Jiao Tong University School of Medicine, Shanghai 200025, China; sj_yyh@sjtu.edu.cn (Y.Y.); dingyilan@sjtu.edu.cn (Y.D.); 2 Shanghai National Clinical Research Center for Endocrine and Metabolic Diseases, Key Laboratory for Endocrine and Metabolic Diseases of the National Health Commission of the PR China, Shanghai National Center for Translational Medicine, Ruijin Hospital, Shanghai Jiao Tong University School of Medicine, Shanghai 200025, China

**Keywords:** microbiota dysbiosis, type 2 diabetes mellitus, metabolism, microbial regulation

## Abstract

Type 2 diabetes mellitus (T2DM) is a common metabolic disorder characterized by insulin resistance and pancreatic β-cell dysfunction. Emerging evidence indicates that gut microbiota dysbiosis may contribute to the development of T2DM. Individuals with T2DM exhibit notable changes in gut microbiota composition, including shifts in the balance between Firmicutes and Bacteroidetes, a reduction in butyrate-producing bacteria, and an increase in opportunistic pathogens. Gut microbiota-derived metabolites—such as short-chain fatty acids, bile acids, and amino acids—have been implicated in the pathogenesis of T2DM, highlighting the critical role of host-microbe interactions. In this overview, we discuss the gut microbiota dysbiosis associated with T2DM and explore the molecular links between microbiota-derived metabolites and the pathogenesis of diseases. Additionally, we explore potential therapeutic strategies, including probiotics and dietary interventions, to modulate the gut microbiota and its metabolites, providing insights for future clinical research and the development of novel treatments for T2DM.

## 1. Introduction

Type 2 diabetes mellitus (T2DM) is a common metabolic disease characterized by insulin resistance and dysfunction of pancreatic β-cells [1]. The prevalence of T2DM continues to rise globally and has become a major public health challenge. It was reported that by 2045, the global prevalence of T2DM would exceed 700 million people [2]. If T2DM remains uncontrolled over a prolonged period, it may lead to cardiovascular diseases [3,4], cancers [5], and neurodegenerative disorders [6], thereby imposing a heavy socioeconomic burden. While genetic predisposition, lifestyle factors, and adiposity have long been recognized as contributors to the pathogenesis of T2DM [7,8], growing evidence suggests that the gut microbiota—a dense and diverse community of microorganisms residing in the gastrointestinal tract—plays a critical role in metabolic homeostasis and may influence the development of T2DM [9,10,11].

The human gut harbors a highly diverse microbiome, comprising bacteria, archaea, fungi, viruses, and other microorganisms. Among these, bacteria are predominant, with the four major phyla—Firmicutes, Bacteroidetes, Actinobacteria, and Proteobacteria—accounting for approximately 64%, 23%, 8%, and 3% of the total microbiota, respectively, together constituting 98% of the community [12]. These bacteria are further classified into numerous genera and species that exhibit distinct metabolic properties [13]. The composition of the gut microbiota varies between individuals, influenced by factors such as age, diet, genetics, and environmental exposures [14]. A balanced gut microbiota is essential for maintaining host metabolic functions and overall health, with microbial communities contributing to energy homeostasis, immune modulation, and metabolic regulation [15].

Gut microbiota dysbiosis, typically characterized by reduced microbial diversity and abundance of the microbiome, disrupts the production of microbial metabolites, which play important roles in host metabolism and disease development [16,17,18]. In recent years, emerging research has provided new perspectives for understanding the role of microbiota dysbiosis in T2DM pathogenesis [19,20,21]. A higher α-diversity of the gut microbiome was reported to be linked to lower insulin resistance and reduced prevalence of T2DM, while variations in gut microbial β-diversity were associated with insulin resistance, and an increase in the abundance of 12 specific taxa may reduce insulin resistance and the risk of T2DM by producing butyrate [22]. Alterations in the gut microbiota may already be present in individuals with combined glucose intolerance, highlighting the potential of microbial biomarkers for the early identification of those at high risk for developing T2DM [23]. Mechanistically, gut microbes contribute to T2DM development and metabolic dysregulation through multiple pathways, including disturbed host insulin sensitivity and glucose and energy homeostasis via gut–brain signaling [24,25]. Hence, targeting these microbes might offer strategies to improve metabolic health.

**Figure 1:**
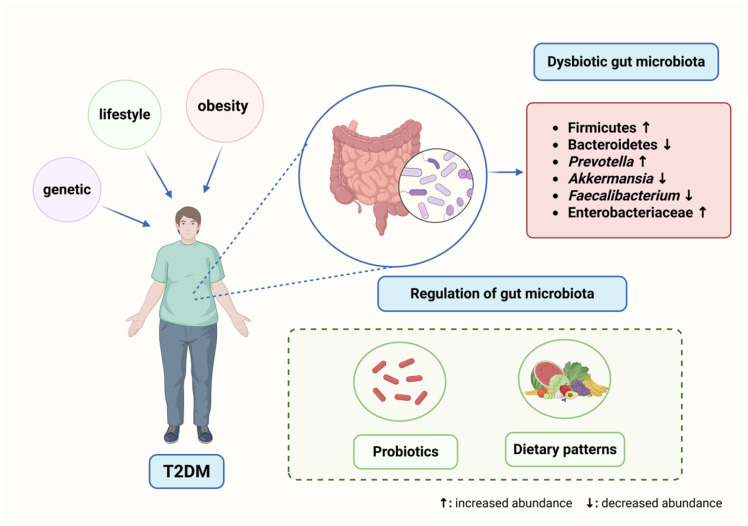
Overview of gut microbiota dysbiosis and regulation in T2DM.

Given the growing evidence, this review discusses characteristic patterns of gut microbiota dysbiosis in T2DM and outlines the mechanisms of dysbiosis-derived metabolites in T2DM. Furthermore, we highlight emerging microbiota-targeted therapeutic strategies, aiming to inspire future clinical research and advance treatment approaches for T2DM (Figure 1).

T2DM is influenced by genetic, lifestyle, and obesity-related factors. Characteristic microbial alterations in T2DM include an increase in Firmicutes, *Prevotella*, and Enterobacteriaceae and a decrease in Bacteroidetes, *Akkermansia*, and *Faecalibacterium*. Gut microbiota can be modulated through probiotics and dietary interventions. This figure was created with https://www.biorender.com/ (accessed on 30 May 2025).

## 2. Gut Microbiota in Healthy Individuals and T2DM

### 2.1. “Healthy” Gut Microbiota

The gut microbiota of healthy individuals exhibits high diversity and dynamic balance, primarily consisting of Firmicutes, Bacteroidetes, Actinobacteria, and Proteobacteria. Among these, Firmicutes and Bacteroidetes dominate, typically maintaining a relatively stable ratio, which is often regarded as an important indicator of gut microbiota health [12]. Microbiota may influence biological processes through multiple mechanisms. Microbiota play a crucial role in extracting energy and nutrients from food because they possess a large number of diverse metabolic genes that encode unique enzymes and metabolic pathways, enabling them to break down complex carbohydrates, proteins, and lipids that the host cannot digest on its own, and generate nutrients that the host can absorb and utilize [12,26,27,28]. Specifically, dietary components influence the abundance of bacteria that produce short-chain fatty acids (SCFAs), which can utilize various substrates (such as sugars, acetate, amino acids, and specific substrates) to synthesize SCFAs. Key enzymes include phosphoenolpyruvate carboxylase (PEPC)/phosphoenolpyruvate carboxylase kinase (PEPCK), malate dehydrogenase (MDH), and thiolase (THL). It has been reported that 74 intestinal bacterial strains produce SCFAs, among which strains belonging to the Firmicutes phylum exhibit the highest species diversity [28]. Bacteroidetes, which primarily includes *Bacteroides*, *Alistipes*, *Parabacteroides*, and *Prevotella*, together with Firmicutes, form the dominant part of the bacterial population in the human gut. Gut-associated Bacteroidetes have the ability to degrade complex polymers, thereby promoting food digestion and nutrient acquisition, particularly in vegetarian and vegan diets [29]. A common feature of the Proteobacteria phylum is Gram-negative staining, indicating the presence of lipopolysaccharides (LPS) in the outer membrane. Many common human pathogens are found in the Proteobacteria phylum. While most microbes in the gastrointestinal tract are obligate anaerobes, Proteobacteria are predominantly facultative anaerobes. In the neonatal gut, where oxygen is relatively abundant, facultative anaerobes such as *Escherichia*, *Klebsiella*, and *Enterobacter* from the Proteobacteria phylum are typically the dominant species during the first week of life. However, these facultative anaerobes are eventually replaced by obligate anaerobes, particularly Firmicutes and Bacteroidetes, which make up the majority of the gut microbiota in healthy adults. A chronic enrichment of Proteobacteria in the gut can represent an imbalanced, unstable microbial community structure or a state of disease of the host [27]. Given the crucial role of the microbiota in human health and its active involvement in various biological processes and disease development, research has gone beyond simply cataloging the composition and correlations of microorganisms. Especially with the emergence of advanced technologies such as high-throughput sequencing, further research is needed to fully understand the functional roles of the human microbiome, which is crucial for promoting microbiome-based diagnosis and personalized medicine. In the context of complex metabolic diseases such as T2DM, this demand is particularly urgent.

### 2.2. Gut Microbiota Dysbiosis in T2DM

#### 2.2.1. Compositional Shifts in the Gut Microbiota of T2DM Patients

T2DM patients exhibit a moderate level of gut microbial dysbiosis, primarily evident at the phylum and class levels [19,30]. However, controversy has been aroused over the changes at the phylum level in individuals with T2DM and prediabetes, indicating uncertainty in this research area. Most studies have found that in individuals with prediabetes and T2DM, there is an increase in the relative abundance of Firmicutes and a decrease in Bacteroidetes, leading to an elevated Firmicutes/Bacteroidetes (F/B) ratio, which is associated with insulin resistance [31,32,33]. However, some studies have found a decrease in Firmicutes and a reduction in the F/B ratio, which may be attributed to variations in population and diet [19,34].

A recent multi-country cohort analysis of 8117 metagenomes revealed strain-specific gut microbial signatures in T2DM. The study identified 5 species associated with T2DM and 14 species associated with both prediabetes and T2DM. Among these, three species were enriched in T2DM: *Clostridium citroniae*, *Clostridium bolteae*, and *Escherichia coli*, while two species, *Coprococcus eutactus* and *Turicibacter sanguinis*, were depleted in T2DM. Most species exhibited a consistent increase or decrease in abundance across normoglycemic controls, prediabetic individuals, and T2DM patients. The study suggested that change in the gut microbiome functioned as a precursor to the onset of T2DM, and further prospective and intervention studies are needed to confirm this (as shown in Table 1) [35]. Compared to healthy controls, Asian individuals with T2DM had higher levels of *Bifidobacterium*, *Streptococcus*, and *Prevotella* and lower levels of *Bacteroides*, *Faecalibacterium*, and *Blautia* at the genus level [36]. In the American population, patients with T2DM also exhibited elevated levels of *Prevotella*. However, these patients primarily have higher levels of *Bacteroides*, *Faecalibacterium*, and *Blautia* than healthy individuals, in contrast to the Asian population. Additionally, *Alistipes* was the genus found at a lower abundance in these patients [20]. Another Finnish cohort found four species consistently associated with incident diabetes, namely *Clostridium citroniae*, *Clostridium bolteae*, *Tyzzerella nexilis*, and *Ruminococcus gnavus.* Notably, two of these species overlap with those reported in the multi-country analyses mentioned above [10,35]. Patients with T2DM are often comorbidly obese. A study from Germany showed that gut microbiota changes were more subtle in obese individuals with T2DM, with only a slight increase in *Escherichia* and *Shigella* compared to non-diabetic obese patients. However, the abundance of *Akkermansia*, *Faecalibacterium*, *Oscillibacter*, and *Alistipes* is significantly reduced in non-diabetic obese individuals. These differences highlight distinct microbial patterns that may help distinguish obesity-associated T2DM from simple obesity [21]. It is worth noting that these changes are not entirely consistent across all populations. Host characteristics such as diet, genetic background, and environmental factors play an important regulatory role.

#### 2.2.2. Functional Shifts in the Gut Microbiota of T2DM Patients

Alongside the changes in bacterial composition, notable alterations of microbial functions were also observed in individuals with T2DM. Numerous studies have consistently reported that T2DM patients show reduced butyrate-producing bacteria and increased opportunistic pathogens [22,30,37]. A Swedish population-based study focused on the gut microbiota profiles of individuals with prediabetes and T2DM. The study consistently found, in both discovery and validation cohorts, that individuals with T2DM had reduced levels of *Clostridium thermocellum*, *Peptoniphilus* sp. *Pral taxon 375*, *Heliobacterium modesticaldum*, *Syntrophobotulus glycolicus*, and *Clostridium* sp. *L2-50*, while the abundance of *Sporosarcina newyorkensis* was increased. In individuals with prediabetes, the levels of *Clostridium ljungdahlii*, *Clostridiales genomosp. BVAB3*, *Clostridium* sp. *BNL1100*, *Desulfosporosinus orientis*, *Heliobacterium modesticaldum*, *Syntrophobotulus glycolicus*, and *Clostridium leptum* were found to be decreased, while the abundance of *Gemella haemolysans* was increased. Most of these decreased bacterial taxa belong to the phylum Firmicutes. And nearly half of the altered metagenomic species in T2DM patients were potential butyrate producers (e.g., *Faecalibacterium*, *Clostridium*, and *Akkermansia*). Another study from the Dutch population also found that a higher abundance of 12 butyrate-producing gut bacteria may benefit the risk of insulin resistance and T2DM [22]. These microbes are generally involved in the breakdown of cellulose and complex carbohydrates, as well as in the production of SCFA. This suggests a potential disruption in energy production and SCFA generation within the gut microbiome, which aligns with the altered metabolic state observed in individuals with prediabetes and T2DM. Compared to healthy individuals, prediabetic and T2DM patients have a significantly higher proportion of conditionally pathogenic bacteria (e.g., Enterobacteriaceae, Desulfovibrionaceae). Hexa-acylated LPS and other Enterobacteriaceae-derived molecules, such as extracellular adhesions and flagellins, can trigger pro-inflammatory pathways via both innate and adaptive immune responses [30,37,38]. In addition, gut microbiota showed increased oxidative stress resistance and reduced functions in flagellar assembly and riboflavin metabolism in both subclinical and clinical statuses of T2DM [39].

**Table 1:** Altered gut microbiota in T2DM across various studies.

Reference	Year of Study	Population	Subjects Sample	Gut Microbiota	Abundance Compared to Control Subjects
Nadja Larsen et al. [19]	2010	Denmark	T2DM (n = 18), control group (n = 18)	Bacteroidetes, Proteobacteria, Bacilli, *Lactobacillus*, *Prevotella* spp.	increase
				Firmicutes, *Clostridia* spp.	decrease
Zhendong Mei et al. [35]	2024	America, Europe, Israel, and China	T2DM (n = 1851), prediabetes (n = 2770), normoglycemic (n = 2277)	*Clostridium citroniae*, *Clostridium bolteae*, *Escherichia coli*, *Streptococcus parasanguinis*, *Streptococcus salivarius*, *Bacteroides fragilis*,	increase
				*Coprococcus eutactus*, *Turicibacter sanguinis*, *Ruminococcus lactaris*, *Bacteroides plebeius*, *Butyrivibrio crossotus*	decrease
Xuangao Wu et al. [36]	2022	Asian (China, India, Japan, Thailand)	T2DM individuals (n = 551), healthy controls (n = 3378)	*ET-L: Escherichia fergusonii*, *Collinsella aerofaciens*, *Streptococcus vestibularis*, *Bifidobacterium longum* *ET-P: Escherichia fergusonii*, *Megasphaera elsdenii*, *Oscillibacter valericigenes*	increase
				*ET-L: Phocaeicola vulgatus*, *Bacteroides uniformis*, *Faecalibacterium prausnitzii* *ET-P: Bacteroides koreensis*, *Faecalibacterium prausnitzii*	decrease
Sunmin Park et al. [20]	2023	American	T2DM individuals (n = 1911), healthy controls (n = 872)	*Enterocloster bolteae*, *Facalicatena fissicatena*, *Clostridium symbiosum*, *Faecalibacterium prausnitzii*	increase
				*Bacteroides koreensis*, *Oscillibacter ruminantium*, *Bacteroides uniformis*, *Blautia wexlerae*	decrease
Matti O Ruuskanen et al. [10]	2022	Finnish	Incident T2DM (n = 432), cohort (n = 5572, 15.8 follow-up years)	*Clostridium citroniae*, *Clostridium bolteae*, *Tyzzerella nexilis*, *Ruminococcus gnavus*	increase
				two *Alistipes* spp.	decrease
Gertraud Maskarinec et al. [9]	2021	White, African American, Native Hawaiian, Japanese American, and Latino	T2DM (n = 307), normoglycemic participant (n = 735), prediabetes (n = 506), undiagnosed T2DM (n = 154)	*Escherichia-Shigella*, Lachnospiraceae	increase
				Actinobacteria, Firmicutes, *Clostridium sensu stricto 1*, *Lachnospira*, Peptostreptococcaceae	decrease
Hao Wu et al. [23]	2020	Swedish	NGT (n = 523), T2DM (at low risk, n = 226; at high risk, n = 297)	*Clostridium bolteae*, *Clostridium clostridioforme*	increase
				*Faecalibacterium* spp., *Clostridium* spp., *Alistipes* spp., *Pseudoflavonifractor* spp., *Oscillibacter* spp.	decrease
Camila Alvarez-Silva et al. [40]	2021	Denmark and India	T2DM (279 Danish and 294 Indian participants)	*Danish:* Bacteroidaceae, Christensenellaceae, Verrucomicrobiaceae, Desulfovibrionaceae, Rikenellaceae, *Akkermansia*, *Alistipes*, *Bacteroides*	increase
				*India:* Lactobacillaceae, Leuconostocaceae, Burkholderiaceae, Prevotellaceae, Prevotella group 9, *Megasphaera*, *Lactobacillus*, *Achromobacter*	increase
Fredrik H Karlsson et al. [41]	2013	European women	T2DM (n = 53), impaired glucose tolerance (IGT; n = 49), normal glucose tolerance (NGT; n = 43)	Clostridiales, *Clostridium clostridioforme*, *Lactobacillus gasseri*, *Streptococcus mutans*	increase
				*Roseburia*, *unidentified Clostridium species*, multiple Clostridiales, *Eubacterium eligens*, Coriobacteriaceae, *Bacteroides intestinalis*	decrease
Afshan Saleem et al. [42]	2022	Pakistanis	T2DM (n = 94)	Lactobacillaceae, Coriobacteriaceae, *Libanicoccus*, *Lactobacillus*, *Collinsella*, *Senegalimassilia*, *Bifidobacterium*, *Slackia*, *Collinsella bouchesdurhonensis*, *Collinsella aerofaciens*	increase
				Ruminococcaceae, Prevotellaceae, *Faecalibacterium*, *Oribacterium*, *Faecalibacterium prausnitzii*	decrease

ET-L, enterotype Lachnospiraceae; and ET-P, enterotype Prevotellaceae. T2DM: Type 2 diabetes mellitus; NGT: Normal glucose tolerance; IGT: Impaired glucose tolerance.

At the functional level, T2DM is characterized by a notable shift in microbial metabolic pathways. Metagenomic analyses have revealed a consistent reduction in 46 microbial pathways in both prediabetes and T2DM, including glycolysis and peptidoglycan biosynthesis, which are essential for energy generation and bacterial cell wall integrity. Conversely, 21 pathways show a consistent enrichment, including those involved in fructose and mannose metabolism, the pentose phosphate pathway, and the biosynthesis of branched-chain amino acids (BCAAs) [23]. These changes reflect a microbiome skewed toward sugar metabolism and amino acid synthesis, potentially contributing to hyperglycemia, insulin resistance, and systemic inflammation. Further functional analyses reveal that microbial genes encoding enzymes for glycolysis (e.g., glyceraldehyde-3-phosphate dehydrogenase) and insulin degradation (e.g., pitrilysin) are enriched in T2DM patients, suggesting that microbial metabolism may directly influence host glucose homeostasis and insulin levels. Pathways associated with saturated fatty acid biosynthesis, as well as those involved in the production of pro-inflammatory bacterial components (e.g., peptidoglycan glycosyltransferase; lipid IVA 4-amino-4-deoxy-L-arabinose transferase), are also upregulated in T2DM, further supporting the link between gut microbiota and metabolic inflammation. Importantly, key metabolic functions related to carbohydrate and lipid metabolism, as well as immune modulation, are predominantly encoded by *Escherichia coli* and various *Bacteroides* species [35]. These findings underscore the need for more detailed, strain-specific functional analyses to fully understand the mechanistic contributions of the gut microbiome to T2DM pathogenesis.

## 3. Mechanism of Gut Dysbiosis in T2DM

**Figure 2:**
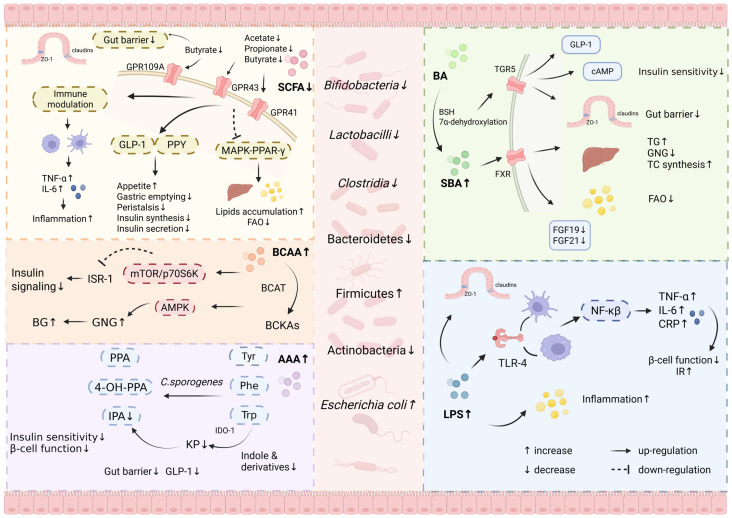
Role of gut dysbiosis-derived metabolites in the pathogenesis of T2DM. The figure depicts four potential pathways of T2DM caused by dysbiosis of the gut flora, including the SCFAs, BAs, AAs, and LPS. This figure was created with https://www.biorender.com/ (accessed on 30 May 2025).

The development of multi-omics technologies has enabled a comprehensive understanding of the pathogenesis of T2DM. Metagenomics facilitates the identification of specific microorganisms associated with T2DM, while metabolomics and proteomics reveal alterations in microbiota-derived metabolites and host interaction networks [43,44]. Although the exact mechanism by which dysbiosis affects T2DM remains unclear, evidence suggests that gut microbe-associated metabolites are important intermediates in the crosstalk between the gut microbiota and the host [45]. Dysregulation of microbe-related metabolites due to dysbiosis, including SCFAs, bile acids (BAs), and endotoxins such as LPS, has an important impact on the development of T2DM [46,47,48,49]. Through modulating gut barrier integrity, immune responses, host metabolic pathways, and gut–brain–pancreas signaling axes, these metabolites serve as key molecular mediators linking gut microbial alterations to insulin resistance, chronic inflammation, and pancreatic β-cell dysfunction. In the following sections, we provide a detailed discussion of each category of these metabolites (Figure 2) [50,51,52].

### 3.1. SCFAs

#### 3.1.1. Relationship Between SCFAs and Gut Barrier Function

SCFAs are primarily produced by anaerobic bacteria in the gut microbiota, particularly *Bifidobacteria*, *Lactobacilli*, *Clostridia*, and other fermentative bacteria. In patients with T2DM, the abundance of these SCFA-producing bacteria is often reduced [53]. These microbes ferment non-digestible carbohydrates derived from dietary fibers, generating acetate, propionate, butyrate, and other SCFAs [54,55]. Acetate, propionate, and butyrate typically exist in the colon and feces at a molar ratio of approximately 60:20:20 [56]. Among these SCFAs, butyrate is considered the primary energy source for intestinal epithelial cells [57,58]. Butyrate enhances gut barrier function through multiple mechanisms, including promoting the expression of tight junction proteins (such as claudin-3, claudin-4, and Zonula Occludens-1 (ZO-1)), stabilizing hypoxia-inducible factor-1α (HIF-1α), and increasing the expression of mucins, antimicrobial peptides, and tight junction proteins, thereby protecting the gut barrier. Additionally, it stimulates goblet cells to secrete mucin-2 (MUC2), regulating the thickness of the intestinal mucus layer [59,60,61]. *Faecalibacterium prausnitzii*, an SCFA-producing bacterium, was reported to be reduced in patients with T2DM, leading to disruption of gut barrier integrity, increased intestinal permeability, translocation of endotoxins into the circulation, and ultimately resulting in systemic endotoxemia and chronic inflammation [62]. Consistently observed in global epidemiological studies, individuals with T2DM or impaired glucose tolerance exhibit a distinct gut microbiota profile, marked by reduced diversity and diminished levels of butyrate-producing bacteria. These bacteria include *Clostridiales* sp. *SS3/4*, *Eubacterium rectale*, *Faecalibacterium prausnitzii*, *Roseburia intestinalis*, and *Roseburia inulinivorans* [30,41,63].

#### 3.1.2. Regulation of Insulin Sensitivity by SCFAs

SCFAs predominantly improve glucose homeostasis and insulin sensitivity through three G-protein-coupled receptors (GPCRs) in the human gut: GPR41, GPR43, and GPR109A. GPR41 and GPR43 are most potently activated by propionate, followed by butyrate and acetate [64,65], whereas GPR109A is exclusively activated by butyrate [66]. SCFAs may regulate T2DM pathogenesis by binding to GPR43 receptors on colonic L cells, promoting glucagon-like peptide-1 (GLP-1) production, and activating GPR41 to increase peptide YY (PYY) secretion. GLP-1 regulates glucose homeostasis by delaying gastric emptying, promoting insulin synthesis and secretion, increasing hepatic glycogen storage, and reducing hepatic glucose output. PYY mainly reduces food intake, inhibits gastric emptying and secretion, and suppresses intestinal motility and pancreatic secretion [64,67,68]. Additionally, SCFAs reduce peroxisome proliferator-activated receptor gamma (PPAR-γ) expression, thereby enhancing oxidative metabolism in the liver and adipose tissue, which in turn decreases fat accumulation, mitigates hepatic steatosis, and improves insulin sensitivity [69].

#### 3.1.3. Role of SCFAs in Fat Metabolism and Low-Grade Inflammation

Propionate and acetate have been shown to exert anti-obesity effects by modulating lipid metabolism in adipose tissue and the liver. Propionate, by activating the adiponectin (APN)-AMP-activated protein kinase (AMPK)-PPARα signaling pathway, reduces lipid accumulation in hepatocytes [70] and regulates the differentiation and function of adipocytes. It also promotes lipolysis and fatty acid utilization while inhibiting fat storage [71,72]. Acetate is primarily absorbed through the portal vein into systemic circulation, where it acts as a substrate for fatty acid synthesis in the presence of acetyl-CoA synthetase in adipocytes [56]. Furthermore, acetate stimulates the AMPK signaling pathway in the liver, enhancing fatty acid oxidation and thus reducing fat storage [73].

In addition to metabolic benefits, SCFAs modulate immune cell function and reduce chronic low-grade inflammation associated with T2DM in obese patients. This process is initiated by the activation of GPR41 and GPR43, which inhibits macrophage activity and reduces the release of pro-inflammatory cytokines such as tumor necrosis factor-α (TNF-α) and interleukin-6 (IL-6) [74,75]. Notably, butyrate reduces pro-inflammatory cytokine production by inhibiting nuclear factor kappa-B (NF-κB) activation in intestinal cells [56,76]. Furthermore, SCFAs reduce inflammation by regulating the number and signaling of various immune cells [77,78]

### 3.2. BAs

BAs are synthesized in the liver from cholesterol, secreted into the intestine to aid fat digestion, and then reabsorbed into the liver via enterohepatic circulation [79]. Gut bacteria play a critical role in BA conversion, particularly through key enzymes such as 7α-dehydroxylase and 7β-dehydroxylase. These bacterial enzymes convert primary BAs into secondary BAs, significantly altering their types, concentrations, and bioactivity. Gut dysbiosis and specific bacterial species such as *Bacteroides*, *Eggerthella lenta*, and *Enterococcus* can significantly affect BA profiles and functions [17,80,81].

The first step of secondary BA metabolism involves the hydrolysis of the amino acid moiety by bile salt hydrolases (BSHs). Metagenomic analyses have revealed that functional BSHs are present across all major bacterial lineages and archaeal species in the human gut, including members of *Lactobacillus*, *Bifidobacterium*, *Clostridium*, and *Bacteroides*. These enzymes are responsible for the deconjugation of BAs, providing precursors for the production of secondary BAs [82]. Once deconjugated, BAs enter the colon and are primarily converted into secondary BAs through 7-dehydroxylation. The two most common secondary BAs in humans are deoxycholic acid (DCA) and lithocholic acid (LCA), which are produced by bacterial 7-dehydroxylation of cholic acid (CA) and chenodeoxycholic acid (CDCA), respectively. *Clostridium* species, particularly *Clostridium scindens*, play a key role in this process [81,83].

Using ultra-performance liquid chromatography-tandem mass spectrometry (UPLC-MS/MS), a study of serum samples from a Chinese population detected 23 BAs. The analysis revealed that conjugated primary BAs (glycocholic acid, taurocholic acid, glycochenodeoxycholic acid, taurochenodeoxycholic acid, and sulfated glycochenodeoxycholic acid) as well as the secondary BA tauroursodeoxycholic acid were positively associated with the incidence of diabetes [84]. BAs influence metabolic and immune functions by activating receptors that regulate key metabolic pathways, including glucose, lipid, and steroid metabolism. Disturbances in serum BA profiles and signaling are strongly associated with T2DM [84]. A cohort study found that a higher proportion of unconjugated secondary BAs in plasma, especially DCA, is linearly associated with a higher risk of cardiovascular disease (CVD) among people with newly diagnosed T2DM. Furthermore, they identified two genetic variants in farnesoid X receptor (FXR, NR1H4) that were significantly associated with CVD [85]. However, a cross-sectional study analyzing human fecal samples identified glycocholic acid (GCA), taurodeoxycholic acid (TDCA), CA, 7-ketolithocholic acid (7-KLCA), taurochenodeoxycholic acid (TCDCA), tauroursodeoxycholic acid (TUDCA), isolithocholic acid (ILCA), and 7-ketodeoxycholic acid (7-KDCA) as the key BAs distinguishing diabetic patients from healthy individuals [86]. 

#### BAs Regulate Metabolic Pathways via Receptor Activation

Since the early identification of BAs as natural ligands for the orphan nuclear receptor, their role as signaling molecules has been extensively confirmed. BAs are also recognized ligands for other key receptors, including FXR, G-protein-coupled bile acid receptor (GPBAR1, TGR5), pregnane X receptor (PXR), and sphingosine-1-phosphate receptor 2 (S1PR2). These nuclear and membrane receptors are widely expressed across the intestine, liver, and other organs. By activating these diverse receptors in various tissues, BAs regulate crucial physiological processes [87,88,89].

Through binding to the FXR, BAs regulate the metabolism of the liver, intestine, and adipose tissue [88,90]. Research suggests that FXR activation positively influences cholesterol, triglyceride, and glucose homeostasis [91]. Specifically, BAs stimulate FXR to lower triglyceride levels, suppress cholesterol synthesis, and enhance fatty acid β-oxidation while simultaneously improving glucose tolerance and insulin sensitivity. Therefore, the deficiency of FXR leads to insulin resistance and hyperglycemia [92]. Reduced FXR activation promotes hepatic gluconeogenesis and elevates blood glucose levels while also impairing insulin resistance and glycogen production. Additionally, it lowers fibroblast growth factor 19 (FGF19) and FGF21 levels and reduces energy expenditure [47]. Dysbiosis of the gut microbiota may lead to insufficient FXR activation, thus affecting T2DM development and progression.

TGR5, another BA receptor, is widely expressed in the intestine, bile ducts, liver, adipose tissue, and immune cells. Its activation strengthens gut barrier function through epithelial integrity and tight junctions. However, in T2DM, gut microbiota dysbiosis can disturb BA metabolism and indirectly weaken TGR5 signaling. This impairs the intestinal barrier and increases gut permeability. As a result, endotoxins such as lipopolysaccharides can enter the circulation, triggering systemic inflammation, which further contributes to the progression of T2DM [93,94]. In addition, TGR5 can affect the metabolic status of T2DM patients through multiple mechanisms. First, by activating the cyclic adenosine monophosphate (cAMP) signaling pathway, TGR5 enhances insulin secretion and improves insulin sensitivity, thereby lowering blood glucose levels and enhancing glucose tolerance [89,95]. Second, it stimulates the secretion of GLP-1, which further supports glucose and lipid metabolism. Moreover, TGR5 activation reduces hepatic lipid accumulation and decreases plasma levels of triglycerides and non-esterified fatty acids [96,97]. As a receptor for secondary BAs, it improves glycemic control in obese mice, reduces plasma triglycerides in hyperlipidemic patients, and increases overall energy expenditure [98,99]. Dysbiosis typically leads to a reduction in secondary BA levels, which in turn impairs the activation of TGR5, resulting in insulin resistance and elevated blood glucose.

### 3.3. Amino Acids

The Framingham Heart Study (FHS) was among the first to report that elevated levels of BCAAs and aromatic amino acids (AAAs) could predict the future development of T2DM [100]. Building on this, a nationwide cohort study in China further demonstrated that a coordinated shift in circulating amino acid profiles—particularly involving BCAAs and AAAs—was already evident in individuals with normal glucose regulation, well before the onset of overt dysglycemia [101]. These findings suggest that amino acid dysregulation may be an early metabolic signature of diabetes development. These associations are thought to be primarily mediated by gut microbiota-derived metabolites that influence host metabolism, inflammation, and insulin sensitivity.

BCAAs, including leucine, isoleucine, and valine, are essential amino acids that play a crucial role in protein synthesis, energy metabolism, and insulin signaling [102,103]. Multiple studies have shown that plasma BCAA concentrations in patients with T2DM are significantly higher than in healthy individuals [104,105]. Elevated BCAA levels can activate the mechanistic target of the rapamycin (mTOR)-p70 S6 kinase (p70S6K) pathway, inhibiting insulin receptor substrate-1 (IRS-1) phosphorylation and reducing insulin signaling, which impairs glucose transport and leads to insulin resistance. This process interferes with the phosphoinositide 3-kinase (PI3K)–protein kinase B (Akt) pathway and is exacerbated under high-fat dietary conditions, highlighting the strong association between elevated BCAA levels and dietary patterns [106,107,108]. In addition, excessive accumulation of BCAAs also activates the AMPK pathway, which enhances hepatic gluconeogenesis while reducing glucose uptake by peripheral tissues, directly leading to elevated blood glucose levels [109,110,111,112].

Under physiological conditions, gut bacteria, such as *Bacteroides* and Firmicutes, help regulate amino acid metabolism by specific enzymes. During dysbiosis, altered activity of these enzymes may disrupt abnormal BCAA degradation, resulting in excessive accumulation [18,113,114]. BCAAs are primarily catabolized in the intestine, starting with branched-chain amino acid transaminases (BCATs), which reversibly transaminate BCAAs into their respective branched-chain keto acids (BCKAs). BCKAs are then decarboxylated by the multienzyme complex branched-chain keto acid dehydrogenase (BCKDH), generating the corresponding acyl-coenzyme A (acyl-CoA) derivatives that participate in various metabolic processes in the body [115,116]. These bacterial enzymes usually catalyze the first step of amino acid transformation, like transamination (BCAA aminotransferase [117]) or decarboxylation (tryptophan decarboxylation [118]). While most enzymes involved in amino acid metabolism are broadly distributed among gut bacteria, some exhibit more specific distribution patterns. For example, the AAA aminotransferase and arginine deiminase are primarily found in Firmicutes and Actinobacteria, whereas BCAA aminotransferase is exclusively present in Bacteroidetes and Firmicutes [18].

A large metagenomic analysis found that *Prevotella copri* Clade A, enriched in some T2DM patients, harbored enhanced BCAA biosynthetic potential, which was largely absent in other clades. Notably, *P. copri Clade A* enrichment varied by ethnicity, being most prevalent in non-Hispanic whites in Western populations, while multi-clade co-occurrence was common in Chinese, Israeli, and U.S. Hispanic populations [35]. In a Danish cohort of non-diabetic males, *Prevotella copri* and *Bacteroides vulgatus* were identified as the primary species responsible for the increased biosynthesis of BCAAs. Among these, *P. copri* was the strongest driver linking microbial BCAA synthesis in the gut to insulin resistance traits. To investigate causality, Pedersen et al. conducted an experiment in which high-fat diet (HFD)-fed mice, initially low in BCAAs, were repeatedly gavaged with *P. copri* for three weeks. The *P. copri* administration resulted in worsened glucose tolerance, elevated serum BCAA levels, and reduced insulin sensitivity compared to control animals that received a sham gavage [119].

AAAs, including tyrosine, phenylalanine, and tryptophan, are also significantly elevated in T2DM patients and are associated with insulin resistance [120]. Gut microbiota—including *Bacteroides*, *Clostridium*, *Bifidobacterium*, and *Lactobacillus acidophilus*—play a critical role in AAA metabolism. Various gut microbes can convert tryptophan into indole and its derivatives. The formation of indole is catalyzed by the enzyme tryptophanase (TnaA), which is found in many Gram-negative and Gram-positive bacterial species, including *Escherichia coli*, *Clostridium* spp., and *Bacteroides* spp. [121]. Gut microbes produce a variety of tryptophan-derived metabolites through multiple metabolic pathways. For instance, *Lactobacillus* spp. convert tryptophan into indolealdehyde (IAld) and indolelactic acid (ILA) via the enzymes AAA aminotransferase and indolelactic acid dehydrogenase (ILDH). Meanwhile, *Ruminococcus gnavus* metabolizes tryptophan into tryptamine through the activity of tryptophan decarboxylase. *Clostridium sporogenes* utilizes a reductive metabolic pathway to convert phenylalanine, tyrosine, and tryptophan into their corresponding aromatic propionate derivatives—phenylpropionate (PPA), 4-hydroxyphenylpropionate (4-OH-PPA), and indolepropionate (IPA) [122].

A multi-ethnic cohort study of 9180 individuals found that higher circulating levels of tryptophan and several kynurenine pathway metabolites were positively associated with an increased risk of T2DM. In contrast, indolepropionate, a microbial tryptophan catabolite, was inversely associated with T2DM risk. A potential causal variable model suggests that the relationship between indolepropionate and T2DM may indeed be causal in nature [123]. The tryptophan–kynurenine pathway (KP) is considered to be significantly associated with T2D. The gut microbiota influences KP metabolism by regulating the activity of the rate-limiting enzyme indoleamine 2,3-dioxygenase 1 (IDO-1) and the availability of tryptophan. Disruption or depletion of the gut microbiota leads to elevated circulating tryptophan levels, reduced kynurenine levels, decreased KP activity, and lower peripheral serotonin concentrations. The introduction of probiotics (such as *Bifidobacterium infantis*) can restore the kynurenine/tryptophan ratio, highlighting the crucial role of the gut microbiota in regulating the tryptophan metabolic pathway [124].

Different dietary sources and processing methods can affect the metabolism of AAAs. A 6-week HFD suppressed the conversion of tryptophan into serotonin, 5-hydroxy-L-tryptophan, melatonin, and various indole metabolites. HFD also reduced microbial diversity and caused significant shifts in microbiota composition. In contrast, diets rich in wheat bran effectively inhibited the conversion of tryptophan into KP metabolites. These effects were associated with lower levels of fasting glucose, total cholesterol, and triglycerides. Wheat bran consumption also promoted the growth of beneficial bacteria such as *Akkermansia* and *Lactobacillus*, which showed significant correlations with tryptophan-derived indolic metabolites [125]. IPA is a unique tryptophan-derived metabolite produced exclusively by gut microbiota. IPA levels are highly influenced by dietary factors, especially fiber intake, and exhibit significant inter-individual variation. Studies in both animal models and human populations have shown that higher serum IPA concentrations are associated with increased dietary fiber intake, a reduced risk of T2DM, and improved insulin secretion. Diets rich in polyphenols, such as the Mediterranean diet (MD), and supplementation with mulberry leaf extract have been shown to increase levels of IPA. In contrast, ketogenic diets, high-fat diets, fried meat, and Western dietary patterns are associated with reduced IPA levels. Specifically, the consumption of fried meat has been shown to decrease gut microbiota diversity, reduce the abundance of *Lachnospiraceae* and *Flavonifractor*, and consequently lower IPA levels [126]. In the Finnish Diabetes Prevention Study, individuals who eventually developed T2D had significantly lower serum IPA concentrations years before the disease onset compared to those who remained diabetes-free over a 15-year follow-up. Among participants who did not progress to T2D, higher IPA levels were linked to better preservation of beta-cell function [127]. Indole and its derivatives can enhance the intestinal epithelial barrier by upregulating genes responsible for maintaining epithelial cell structure and function [128], stimulate GLP-1 secretion to promote insulin release and suppress appetite, and slow gastrointestinal motility via serotonin (5-HT) production [123,129].

### 3.4. Endotoxin

As a component of Gram-negative bacterial cell walls, LPS can enhance intestinal permeability, contribute to chronic low-grade inflammation in obesity, and drive the progression of T2DM [130,131]. Increased adherence of intestinal *Escherichia coli* and a decrease in intestinal *Bifidobacterium* species are associated with increased serum LPS [132]. Once LPS enters the bloodstream, it binds to Toll-like receptor 4 (TLR4) on macrophages and dendritic cells [133,134]. Activation of TLR4 triggers the immune response and the NF-κB signaling pathway, leading to the release of inflammatory cytokines such as TNF-α, IL-6, and C-reactive protein (CRP). These cytokines directly damage pancreatic β-cells, impair insulin secretion, and induce insulin resistance, thereby accelerating the onset and progression of T2DM [135,136,137]. LPS also promotes macrophage infiltration in adipose tissue, enhances local and systemic inflammation, and accelerates the development of obesity, insulin resistance, and T2DM [137,138]. LPS activates immune and inflammatory pathways, with sustained high levels of LPS promoting fat accumulation and leading to obesity. Inflammatory cytokines released from adipose tissue not only exacerbate fat deposition but also trigger insulin resistance, reducing insulin sensitivity. Furthermore, LPS influences liver fat metabolism, increasing hepatic fat deposition and further aggravating liver insulin resistance [139,140,141].

Metabolic concentrations of plasma LPS are modulated by fat food content [131,142]. Studies have shown that an HFD increases plasma LPS concentrations by two to three times compared to a low-fat, low-carbohydrate control diet or a high-carbohydrate diet. Notably, an HFD promotes the growth of an LPS-enriched microbiota. Among the major gut bacterial groups, the Gram-negative *Bacteroides*-related mouse intestinal bacteria group was significantly reduced in mice fed an HFD compared to controls. Similarly, the dominant Gram-positive group, *Eubacterium rectale–Clostridium coccoides*, as well as bifidobacteria—known for their ability to reduce intestinal LPS levels and enhance mucosal barrier function—also showed reduced abundance in HFD-fed mice [131]. Studies indicate that changes in gut bacteria induced by a high-fat diet significantly enhance intestinal permeability by downregulating the expression of genes encoding the tight junction proteins ZO-1 and occludin. This diet-induced dysbiosis is characterized by a reduction in *bifidobacteria*, which are known to lower intestinal LPS levels and enhance mucosal barrier integrity [130]. *Akkermansia muciniphila*, a species that resides in the intestinal mucus layer, has been shown to upregulate the expression of tight junction proteins, including zonula occludens-1 and occludin [143]. A depletion of *A. muciniphila* may compromise gut barrier integrity, leading to increased translocation of LPS into the bloodstream. Additionally, the postprandial period tends to be prolonged after consuming an HFD, further elevating the rate of LPS translocation [144]. *Lactobacillus rhamnosus* strain GG has been shown to protect epithelial monolayers from enterohemorrhagic *Escherichia coli* (EHEC)-induced redistribution of the tight junction proteins claudin-1 and ZO-1. However, unlike the live probiotic, heat-inactivated *L. rhamnosus GG* had no effect on EHEC attachment, A/E lesion formation, or disruption of the epithelial barrier [145].

Clinical studies and animal models have demonstrated that intermittent fasting (IF) can reduce LPS levels [146,147]. An 8-week clinical trial of modified IF showed significant changes in the gut microbiota, affecting 23 species—10 from Firmicutes and 7 from Proteobacteria. These results align with findings that IF mainly alters Firmicutes. IF also boosted SCFA production and significantly reduced plasma LPS levels [146]. SCFA production enhances the intestinal barrier, limiting LPS translocation into the bloodstream. This process reduces activation of TLR4, leading to lower secretion of pro-inflammatory cytokines such as TNF-α and IL-6, which are involved in insulin resistance [148]. IF tends to increase *Bifidobacterium*, which supports intestinal barrier integrity and microvilli health, and also boosts *Allobaculum*, an SCFA producer. SCFAs promote epithelial metabolism, lower intracellular oxygen, stabilize HIF-1, and strengthen the gut barrier, reducing intestinal permeability. As LPS primarily originates from Enterobacteriaceae, their abundance increased in diet-induced obese mice, but IF reversed this trend. However, this study only investigated one type of IF (every-other-day fasting), so the results may not apply to other fasting regimens [147].

## 4. Potential Therapeutic Strategies Targeting Gut Microbiota

### 4.1. Probiotics and Prebiotics

In recent years, modulation of gut microbiota has emerged as a potential strategy for treating T2DM and related metabolic disorders (as shown in Table 2). Among the various approaches, probiotic or prebiotic supplementation is considered simple yet effective [149,150]. Evidence from both animal and clinical studies has shown that administration of probiotics, such as *Bifidobacterium*, *Lactobacillus*, and *Akkermansia*, is beneficial for obesity and T2DM [11,151,152]. In high-fat diet (HFD)-induced obese mice, probiotics help restore intestinal barrier function and reduce fat tissue and intestinal inflammation. The mice experience reduced activation of pro-inflammatory signaling pathways, such as the TLR4/NF-κβ axis. Additionally, the expression of inflammatory cytokines like TNF-α, IL-1β, and interferon-γ (IFN-γ) decreases, ultimately leading to improved insulin resistance [151]. A meta-analysis including 412 obese patients demonstrated that probiotic supplementation effectively reduces body weight, waist circumference, and visceral fat content in obese individuals [153]. In db/db mice, multi-strain probiotic supplementation enhanced the abundance of SCFA-producing bacteria and improved intestinal barrier function [154,155]. A randomized controlled trial of 88 obese T2DM patients showed that 24 weeks of synbiotic supplementation modulated the gut microbiota by increasing the abundance of *Bifidobacteria* and *Lactobacilli*, as well as the concentration of fecal organic acids [152].

Different *Lactobacillus* species may have varying effects on blood glucose levels in patients with T2DM. In a double-blind trial, 12 weeks of oral supplementation with *Lactobacillus reuteri* DSM 17938 in patients with T2DM improved insulin sensitivity in those with higher microbial diversity at baseline but had no effect on HbA1c [170]. In another randomized, double-blind, placebo-controlled trial involving 68 T2DM patients, a significant reduction in HbA1c was observed in participants who consumed *L. reuteri* strains ADR-1 and ADR-3, provided their fecal *L. reuteri* increased by at least 8-fold. The study also found that different *L. reuteri* strains influenced gut microbiota differently. In the ADR-1 group, fecal *Lactobacillus* levels were positively correlated with *Bifidobacterium* but negatively with Bacteroidetes. In the ADR-3 group, fecal *L. reuteri* abundance was positively associated with Firmicutes [171]. The intake of different *L. reuteri* strains may affect gut microbiota composition differently, potentially leading to varying outcomes after probiotic use. Supplementation with other *Lactobacillus* species has also shown potential effects on glycemic parameters in T2DM patients. In a 90-day randomized clinical trial involving non-diabetic middle-aged and older adults, supplementation with *Lactobacillus rhamnosus* GG helped stabilize HbA1c levels, while HbA1c significantly increased in the placebo group. These results suggest that probiotics may offer potential benefits for glycemic control [172]. In a double-blind RCT with 45 men of varying glucose tolerance, 4 weeks of supplementation with *Lactobacillus acidophilus* NCFM helped preserve insulin sensitivity compared to the placebo group [173]. The beneficial effects of *Lactobacillus* and *Bifidobacterium* on T2DM can be attributed to their ability to modulate signaling pathways such as NF-κB, PI3K/Akt, and nuclear factor erythroid 2–related factor 2 (Nrf2), leading to significant improvements in glucose metabolism and insulin resistance [174].

Prebiotic-driven modulation of the gut microbiota also increases the endogenous production and portal vein secretion of gut peptides like GLP-1 and PYY from enteroendocrine L-cells (L-cells) [175]. Compared to metformin alone, the addition of probiotics significantly lowers fasting blood glucose and hemoglobin A1c (HbA1c) levels in T2DM patients. This highlights the potential of probiotics as an adjunctive therapy. Targeted probiotic supplementation may offer benefits for individuals who respond poorly to standard medications [176]. Recently, a clinical trial from China indicates the efficacy of *Akkermansia muciniphila* supplementation in overweight/obese T2DM depends on its baseline abundance. Patients with low baseline bacteria levels of *A. muciniphila* exhibited greater reductions in body weight, fat mass, and blood glucose levels. This highlights the potential of personalized gut microbiota modulation as a promising strategy for treating T2DM [11].

A meta-analysis of 33 clinical trials found that supplementation with inulin-type fructans significantly reduced FBG, HbA1c, fasting insulin, and HOMA-IR in individuals with prediabetes and T2DM. Based on these findings, the study recommends a daily intake of 10 g for a minimum of six weeks [177]. Zhang et al. evaluated the effects of probiotics on glycemic markers in T2DM. The results showed that multi-strain and high-dose probiotics had stronger benefits for blood glucose regulation compared to single-strain and low-dose probiotics. Specifically, greater reductions in fasting glucose and HOMA-IR were observed with higher probiotic doses, suggesting a dose–response relationship. The effectiveness of probiotics also depended on the strain type. For instance, *brewer’s yeast* significantly lowered fasting glucose, while *Lactobacillus casei* and *Lactobacillus sporogenes* showed no effects when used alone. However, probiotic supplementation did not significantly affect HbA1c levels [164]. A meta-analysis of 46 RCTs evaluated the effectiveness of probiotics and synbiotics in prediabetes and T2DM. The results showed that both probiotics and synbiotics improved glycemic markers, lowering FBG, HbA1c, fasting insulin, and HOMA-IR, while increasing QUICKI. However, the improvements in FBG, HbA1c, and HOMA-IR were more pronounced in patients who consumed probiotics compared to those who took synbiotics [157]. Another study suggests that synbiotics may offer added benefits over probiotics alone for improving insulin levels, with a moderate but statistically significant effect size. However, results showed high variability, indicating that factors such as strain type, dosage, and individual differences may influence the effectiveness of probiotics and synbiotics in diabetes management. Overall, the therapeutic effects of these interventions appear to be strain-specific, highlighting the need for further research on the distinct roles of individual strains [161]. 

### 4.2. Impact of Dietary Interventions on Gut Microbiota

Diet is a major determinant of gut microbiota composition, and different dietary patterns exert distinct influences on microbial diversity and metabolic function. These microbiota-mediated effects are increasingly recognized as key modulators of T2DM pathophysiology [178,179,180].

A high-fiber diet, in particular, has been shown to positively influence gut microbiota. These beneficial bacteria ferment the fibers to produce SCFAs, which enhance the gut environment and inhibit the growth of harmful microbes. SCFAs not only strengthen the intestinal barrier but also regulate fat metabolism and improve insulin sensitivity, thereby aiding in the control of obesity and T2DM [54,181]. Arabinoxylan, a well-known type of hemicellulose, was used in a human intervention study targeting metabolic parameters in overweight individuals, resulting in increased abundance of *Prevotella* and *Eubacterium rectale* [182]. Dietary supplementation with oat-derived β-glucan elevated *Bacteroidetes* levels while reducing *Firmicutes*, and increased whole-grain barley intake has been associated with a higher abundance of *Prevotella copri* [178,183].

In animal studies, resistant starch (RS) showed antidiabetic effects similar to metformin, including significantly lowering blood glucose, improving insulin resistance and glucose tolerance, and alleviating tissue damage in T2DM rats. Both treatments enhanced gut bacterial diversity and restored SCFA-producing bacteria, leading to higher SCFA levels. However, RS was more effective at increasing microbiota diversity, particularly enriching *Prevotella* species [184]. The effects of RS on glycemic control in T2DM patients remain controversial. Lin et al. evaluated the impact of a novel RS formula, PPB-R-203, on glucose homeostasis in both healthy individuals and patients with T2DM. They found that PPB-R-203 significantly improved postprandial blood glucose levels in T2DM patients [185]. However, another study that used type 2 resistant starch (HAM-RS2) for a 12-week intervention found no significant differences in fasting plasma glucose, HbA1c, insulin sensitivity, or beta-cell function, as measured by the HOMA, between the RS2 group and the control group [186]. These findings suggest that fiber-induced microbiota shifts are subtype-specific, necessitating further investigation into the microbial degradation pathways associated with different fiber types [54].

A high-protein diet (accounting for 30% of total energy intake, or ≥1.1 g/kg/day of protein) can help reduce hyperglycemia in patients with T2DM, in part due to its BCAA content [187]. The BCAA content of foods varies by source, whether animal or plant. Both sources are important, but the type and amount of BCAAs can lead to different metabolic responses. In T2DM patients, animal protein, compared to plant protein at the same calorie level, increases postprandial BCAA levels more and has a greater effect in reducing liver fat [188]. Recent data from large cohorts have also confirmed the association between animal or plant protein and T2DM. Total protein and animal protein may increase the risk of T2DM, while plant protein shows no association or is inversely related to the risk [189,190]. The exact biological mechanisms underlying the different associations between animal and plant protein intake and T2DM risk remain unclear. These differences may relate to the food sources, other nutrients present in protein-rich foods, and variations in amino acid composition. Red and processed meat intake has been linked to a higher risk of T2DM. Adjusting for BCAAs and AAAs weakens the association between protein intake and T2DM risk, suggesting they may partly mediate this relationship [189]. A diet rich in plant-based protein should be considered to replace animal protein for the prevention of diabetes.

The use of low-carbohydrate diets to manage diabetes dates back to the pre-insulin era. Contemporary evidence shows that such diets effectively reduce fasting blood glucose and show a significant decrease in HbA1c levels [191]. Additionally, such diets enhance glucose metabolism in T2DM patients by sustaining GLP-1 secretion [179,192]. It also influences gut microbiota composition by promoting the proliferation of beneficial bacteria, such as Firmicutes and Bacteroidetes, while inhibiting the growth of pathogenic microbes. These changes may further influence T2DM progression through the gut–brain axis [193]. The degree of carbohydrate restriction also matters. One study comparing the benefit role of a low-carbohydrate ketogenic diet (LCKD) with the low-calorie diet (LCD) among T2DM patients found that during the 6-month dietary intervention, LCKD led to greater improvements in glycemia, body weight, body mass index, blood glucose, total cholesterol, low-density lipoprotein (LDL) cholesterol, triglycerides, and urea levels [194]. Similarly, a one-year intervention in overweight adults with T2DM or prediabetes showed that participants on an LCKD had greater reductions in HbA1c and required fewer diabetes medications than those on a moderate-carbohydrate diet [195]. Shotgun metagenomics of cecal contents from mice fed an LCKD revealed a significant drop in α-diversity, which was restored with methionine supplementation. LCKD also decreased the relative abundance of Firmicutes, Actinobacteria, and Verrucomicrobia, while increasing Proteobacteria. Methionine supplementation reversed these changes. Four key strains—*Lactobacillus murinus* ASF361, *Lactobacillus reuteri* (unclassified), *Akkermansia muciniphila* ATCC BAA-835, and *Helicobacter hepaticus* ATCC 51449—were most affected by LCKD and recovered with methionine. These strains were significantly linked to fasting blood glucose and may mediate metabolic effects by influencing serum levels of TDCA and TUDCA [196]. In a study of 12 children with therapy-resistant epilepsy treated with a 3-month LCKD, a significant decrease in the relative abundance of Bifidobacteria, *Eubacterium rectale*, and *Dialister* was observed. Meanwhile, the relative abundance of *Escherichia coli* increased during the intervention [197]. These changes result in lower production of SCFAs, especially acetate and butyrate, which play crucial roles in supporting intestinal barrier function and immune balance. Reduced SCFA levels may increase the risk of intestinal inflammation and vulnerability to infections by pathogens [197,198].

The MD, characterized by high intake of monounsaturated fatty acids, dietary fibers, and antioxidants, has also been shown to beneficially reshape gut microbiota [180,199]. Obese and overweight individuals who follow the MD exhibit an increase in the fiber-degrading bacterium *Faecalibacterium prausnitzii* and enhanced expression of microbial carbohydrate degradation genes associated with butyrate metabolism [180,200]. Furthermore, in T2DM patients, MD intervention can beneficially improve the gut microbiota composition, particularly by increasing the abundance of Firmicutes, Bacteroidetes, and Bifidobacteria [166]. These changes are associated with BA degradation and urolithin production, as well as improvements in fat metabolism and insulin sensitivity, underscoring the critical role of gut microbiota alterations in regulating lipid and glucose metabolism [200,201,202].

Other external factors—such as antidiabetic medications, smoking, artificial sweeteners, and food additives—may confound the relationships among diet, microbiota, and T2DM. Forslund et al. found that metformin, a common diabetes medication, alters the gut microbiota in T2DM patients. T2DM patients on metformin had more *Escherichia* spp. and fewer *Intestinibacter* spp. Compared to untreated patients. Since *Escherichia* spp. can produce SCFAs like butyrate and propionate, which stimulate intestinal gluconeogenesis and lower blood glucose, the findings suggest that metformin’s glucose-lowering effects are partly mediated by gut microbiota [203]. In Japanese patients with T2DM, acarbose treatment led to a significant increase in *Bifidobacterium* and *Lactobacillus* abundance, while *Bacteroides* decreased. *Bifidobacterium* species can produce formate and acetate under low-carb conditions and lactate and acetate when carbohydrates are abundant. Other gut microbes then convert lactate into butyrate and propionate. Thus, the acarbose-induced rise in *Bifidobacterium* may promote SCFA production in the gut of T2DM patients [204]. In T2DM patients, smoking is linked to dietary choices and gut microbiota changes. Current smokers tend to consume more alcohol, sugar, and sweeteners, and less fruit. Their gut microbiota shows a higher proportion of *Coprococcus* at the genus level [205]. Sweeteners in both mice and humans can affect glucose tolerance [206]. A meta-analysis examined the effects of common sweeteners on the gut microbiota and found that artificial sweeteners may contribute to metabolic issues like obesity and T2DM. Among nonnutritive sweeteners (NNSs), only saccharin and sucralose significantly alter gut microbiota. Saccharin reduced the growth of six bacterial strains (three *Lactobacillus* and three *Escherichia coli*) in rats, while sucralose decreased total counts of anaerobes, aerobes, bifidobacteria, lactobacilli, *Bacteroides*, and *Clostridium*. For nutritive sweeteners, stevia was the only one shown to affect gut microbiota. Some polyols, like isomalt and maltitol, increased *Bifidobacterium* in healthy subjects, suggesting a prebiotic effect. However, lactitol reduced *Bacteroides*, *Clostridium*, coliforms, and *Eubacterium* while increasing butyrate and IgA without causing gut inflammation, showing a symbiotic effect. Xylitol reduced fecal Bacteroidetes and *Barnesiella*, increased Firmicutes and *Prevotella*, and influenced *C. difficile* in mice [207]. Certain factors that contribute to harmful metabolic effects, such as the consumption of non-caloric artificial sweeteners and emulsifiers (commonly used as additives in processed foods), can also impair glycemic control and promote the overgrowth of Proteobacteria [206]. Notably, the increase in the relative abundance of the family Enterobacteriaceae and the class Deltaproteobacteria induced by artificial sweeteners aligns with findings from patients with T2DM [30].

## 5. Conclusions and Future Perspectives

Over the past decades, rapid advances in multi-omics technologies have greatly enhanced our understanding of the complex interactions between gut microbes and the host. Nevertheless, our knowledge remains limited regarding their precise roles in disease onset, underlying mechanisms, and pathophysiology. Although existing studies have preliminarily elucidated the potential mechanisms linking intestinal dysbiosis and T2DM, several key scientific challenges persist. First, the causal relationship between gut microbiota dysbiosis and T2DM has yet to be conclusively established. Longitudinal cohort studies and animal models are needed to verify the temporal sequence and establish causality. Second, significant differences in gut microbiota composition across different populations (e.g., ethnicity, dietary structure) suggest the need to establish personalized flora intervention strategies in the future. Furthermore, different strains within the same genus (e.g., *E. coli* commensals versus pathogenic strains) may have completely opposite functional properties, requiring research tools to move toward higher-resolution macrogenomic or culture genomics techniques.

Compared with traditional antidiabetic therapies, microbiota-targeted modulation offers several unique advantages. It facilitates a shift from standardized treatment to personalized therapeutic strategies. When used adjunctively, it may allow for dose reduction in traditional medications and minimize long-term adverse effects. Furthermore, it confers multiple metabolic benefits, including improvements in insulin sensitivity and systemic metabolic homeostasis, while also helping prevent diabetes-related complications.

Despite growing evidence linking specific microbial taxa to T2DM pathophysiology, only a limited number have been explored as therapeutic agents. Future research should prioritize well-designed, large-scale clinical trials to validate promising microbial targets and facilitate the development of effective, personalized microbiota-based interventions.

## Figures and Tables

**Table 2:** Gut microbiota intervention in patients with T2DM.

References	Year	Intervention	Genus	Study Type	RCT	Patients	Sample Size	Main Results (Intervention Group vs. Control Group)
Paul et al. [156]	2022	Probiotics, prebiotics, and synbiotics	Probiotics: *Lactobacillus*, *Bifidobacterium*, *Streptococcus*, *Lactococcus*, *Bacillus*, *Acetobacter*, *Propionibacterium*, *Akkermansia*, *Clostridium*, *amd Anaerobutyricum*. Prebiotics: inulin/FOS, GOS, resistant dextrin, resistant starch, beta-glucan, and mixed/complex prebiotics.	Meta-analysis	68 RCTs	T2DM	n = 3835	fasting glucose ↓ HbA1c ↓ fasting insulin ↓ HOMA-IR ↓ QUICKI ↑
Naseri et al. [157]	2022	Probiotics and synbiotics	/	Meta-analysis	46 RCTs	T2DM	n = 3067	FPG ↓ HbA1c ↓ fasting insulin ↓ HOMA-IR ↓ QUICKI ↑
Rittiphairoj et al. [158]	2021	Probiotics	Probiotics: *Lactobacillus*, *Bifidobacterium*, *Streptococcus*, *Lactococcus*, *Bacillus*, *Propionibacterium*, *Acetobacter*, *and Enterococcus.*	Meta-analysis	28 RCTs	Prediabetes/T2DM	n = 1947	FPG ↓
Li et al. [159]	2023	Probiotics	Probiotics: *Lactobacillus*, *Bifidobacterium*, *Streptococcus*, *Lactococcus*, *Bacillus*, *Saccharomyces*, *Propionibacterium*, *and Acetobacter.*	Meta-analysis	30 RCTs	T2DM	n = 1827	FPG ↓ HbA1c ↓ fasting insulin ↓ HOMA-IR ↓
Tabrizi et al. [160]	2017	Synbiotic	*Lactobacillus*, *Bifidobacterium.*	Meta-analysis	7 RCTs	T2DM/GDM	n = 482	FPG ↓ insulin ↓ HOMA-β ↓ QUICKI ↑
Baroni et al. [161]	2024	Probiotics/synbiotics	Probiotics: *Lactobacillus*, *Bifidobacterium*, *Streptococcus*, *Lactococcus*, *Bacillus*, *Clostridium*, *Akkermansia*, *Anaerobutyricum*, *Propionibacterium*, *Acetobacter*, *and Saccharomyces.*	Meta-analysis	41 RCTs	T1DM/T2DM	n = 2991	FPG ↓ HbA1c ↓ fasting insulin ↓
Jayedi et al. [162]	2024	Probiotics, prebiotics, and synbiotics	Probiotics: *Lactobacillus*, *Bifidobacterium*, *Streptococcus*, *Propionibacterium*, *and Bacillus.*	Meta-analysis	68 RCTs	T2DM	n = 4249	FPG ↓ HbA1c ↓
Xiao et al. [163]	2023	Probiotic	Probiotics: *Lactobacillus*, *Bifidobacterium*, *Streptococcus*, *Propionibacterium*, *Acetobacter*, *and Saccharomyces.*	Meta-analysis	37 RCTs	T2DM	n = 2502	FPG ↓ HbA1c ↓ fasting insulin ↓ HOMA-IR ↓
Zhang et al. [164]	2022	Probiotics	/	Meta-analysis	33 RCTs	T2DM	n = 1927	FPG ↓ HbA1c ↓ fasting insulin(-) HOMA-IR ↓
Bock et al. [165]	2021	Probiotics, prebiotics, or synbiotics	Probiotics: *Lactobacillus*, *Bifidobacterium*, *Streptococcus*, *Lactococcus*, *Propionibacterium*, *Acetobacter*, *Bacillus*, *and Saccharomyces.*	Meta-analysis	38 RCTs	T1DM/T2DM	n = 2086	FPG ↓ HbA1c(-) fasting insulin ↓
Dimba et al. [166]	2024	Prebiotics/Mediterranean/plant-based diet	/	Meta-analysis	8 RCTs	Prediabetes/T2DM	n = 488	Prebiotics:FPG(-), HbA1c(-) MD:FPG ↓ HbA1c ↓ Plant-Based Diet:FPG(-),HbA1c(-)
Ojo et al. [167]	2020	Dietary fiber	*Bifidobacterium* ↑	Meta-analysis	9 RCTs	T2DM	n = 704	HbA1c ↓ FPG(-) HOMA-IR(-)
Houghton et al. [168]	2018	Synbiotic supplementation, strict vegetarian diet, Ma-Pi diet, Type 2 diabetes diet with increased sardine intake, probiotic supplementation, prebiotic supplementation, digestive supplement	No specific genus change, but altered gut microbiota diversity and structure.	Meta-analysis	8 RCTs	T2DM	n = 395	HbA1c ↓ FPG(-), fasting insulin(-), HOMA-IR(-)
Ojo et al. [169]	2021	Almonds	Promoting short-chain fatty acid-producing bacteria	Meta-analysis	8 RCTs	T2DM	n = 221	HbA1c ↓ FBG(-), fasting insulin(-), HOMA-IR(-)

HbA1c, Glycated Hemoglobin A1c; HOMA-IR, Homeostasis Model Assessment of Insulin Resistance; QUICKI, Quantitative Insulin Sensitivity Check Index; FPG, Fasting Plasma Glucose; FBG, Fasting Blood Glucose; HOMA-β, Homeostasis Model Assessment of Beta-cell Function; MD, Mediterranean diet. ↑, increase; ↓, decrease; (-), no significant change.

## Data Availability

No new data were created or analyzed in this study.

## Abbreviations

Type 2 diabetes mellitus (T2DM); short-chain fatty acids (SCFAs); phosphoenolpyruvate carboxylase (PEPC); phosphoenolpyruvate carboxylase kinase (PEPCK); malate dehydrogenase (MDH); thiolase (THL); lipopolysaccharides (LPS); branched-chain amino acids (BCAAs); bile acids (BAs); fatty acid oxidation (FAO); gluconeogenesis (GNG); blood glucose (BG); tyrosine (Tyr); phenylalanine (Phe); tryptophan (Trp); *Clostridium sporogenes* (C. sporogenes); triglyceride (TG); total cholesterol (TC); branched-chain amino acid transaminase (BCAT); Zonula Occludens-1(ZO-1); hypoxia-inducible factor-1α (HIF-1α); mucin-2 (MUC2); G-protein-coupled receptors (GPCRs); glucagon-like peptide-1 (GLP-1); Peptide YY (PYY); peroxisome proliferator-activated receptor gamma (PPAR-γ); adiponectin (APN); AMP-activated protein kinase (AMPK); tumor necrosis factor-α (TNF-α); interleukin-6 (IL-6); nuclear factor kappa-B(NF-κB); bile salt hydrolases (BSHs); deoxycholic acid (DCA); lithocholic acid (LCA); cholic acid (CA); chenodeoxycholic acid (CDCA); ultra-performance liquid chromatography-tandem mass spectrometry (UPLC-MS/MS); cardiovascular disease (CVD); Farnesoid X Receptor (FXR, NR1H4); glycocholic acid (GCA); taurodeoxycholic acid (TDCA); 7-ketolithocholic acid (7-KLCA); taurochenodeoxycholic acid (TCDCA); tauroursodeoxycholic acid (TUDCA); isolithocholic acid (ILCA); 7-ketodeoxycholic acid (7-KDCA); G-protein-coupled bile acid receptor, GPBAR1(TGR5); pregnane X receptor (PXR); sphingosine-1-phosphate receptor 2 (S1PR2); fibroblast growth factor 19 (FGF19); fibroblast growth factor 21 (FGF21); Cyclic adenosine monophosphate (cAMP); The Framingham Heart Study (FHS); aromatic amino acids (AAAs); mechanistic target of rapamycin (mTOR)/p70 S6 kinase (p70S6K); insulin receptor substrate-1 (IRS-1); Phosphoinositide 3-kinase (PI3K)–protein kinase B (Akt); branched-chain amino acid transaminases (BCATs); branched-chain keto acids (BCKAs); branched-chain keto acid dehydrogenase (BCKDH); acyl-coenzyme A (acyl-CoA); high-fat diet (HFD); tryptophanase (TnaA); indolealdehyde (IAld); indolelactic acid (ILA); indolelactic acid dehydrogenase (ILDH); phenylpropionate (PPA); 4-hydroxyphenylpropionate (4-OH-PPA); indolepropionate (IPA); kynurenine pathway (KP); indoleamine 2,3-dioxygenase 1 (IDO-1); Mediterranean diet (MD); serotonin (5-HT); Toll-like receptor 4 (TLR4); C-reactive protein (CRP); Enterohemorrhagic *Escherichia coli* (EHEC); intermittent fasting (IF); interferon-γ (IFN-γ); nuclear factor erythroid 2–related factor 2 (Nrf2); Enteroendocrine L-cells (L-cells); resistant starch (RS); low-carbohydrate ketogenic diet (LCKD); low-calorie diet (LCD); low-density lipoprotein (LDL); nonnutritive sweeteners (NNSs).
